# Cardiac abnormalities and facial anthropometric measurements in children from the Free State and Northern Cape provinces of South Africa with chromosome 22q11.2 microdeletion

**Published:** 2010-02

**Authors:** SC Brown, DA Buys, BD Henderson, M Theron, MA Long, F Smit

**Affiliations:** Department of Paediatric Cardiology, Faculty of Health Sciences, University of the Free State, Bloemfontein, South Africa; Department of Paediatric Cardiology, Faculty of Health Sciences, University of the Free State, Bloemfontein, South Africa; Division of Human Genetics, Faculty of Health Sciences, University of the Free State, Bloemfontein, South Africa; Division of Human Genetics, Faculty of Health Sciences, University of the Free State, Bloemfontein, South Africa; Department of Cardiothoracic Surgery, Faculty of Health Sciences, University of the Free State, Bloemfontein, South Africa; Department of Cardiothoracic Surgery, Faculty of Health Sciences, University of the Free State, Bloemfontein, South Africa

**Keywords:** cardiac lesion, 22q11 microdeletion, facial anthropometry, echocardiography

## Abstract

**Introduction:**

Microdeletions of chromosome 22 are common and have a prevalence of at least 1/4 000. Cardiac abnormalities, abnormal facial features and palatal abnormalities are frequently present in these patients.

**Aim:**

To describe the cardiac lesions and selected measurable facial features in children from the Free State and Northern Cape presenting at the Cardiology Unit of the Universitas Academic Hospital complex in Bloemfontein.

**Methods:**

This was a prospective study in which patients with abnormal facial characteristics were tested using a fluorescence *in situ* hybridisation (FISH) probe for the 22q11.2 microdeletion. Forty children tested positive for the microdeletion. All patients underwent an echocardiogram and where possible, facial anthropometric measurements were performed.

**Results:**

The median age at diagnosis was 3.6 years (range 0.04 years, i.e. 2 weeks to 16.2 years). Tetralogy with or without pulmonary atresia was diagnosed in 43% (*n* = 17) of the children and truncus arteriosus in 20% (*n* = 8). A rightsided aortic arch was present in 43% (*n* = 17) of the patients. Mid-facial height was slightly longer (median *z* = 1.0; range –0.5 to 3.3) and width narrower (median *z* = –1.4; range –2.2 to 0.1) than normal. Ear height and width were notably small compared to normal, with median *z*-scores = –3.3 (range –4.8 to –2.6) and *z* = –2.4 (range –3.4 to –1.4), respectively.

**Conclusions:**

Microdeletions of chromosome 22q11 are present in children from the Free State and Northern Cape. Conotruncal cyanotic heart lesions, especially tetralogy with or without pulmonary atresia and truncus arteriosus were the most frequent congenital cardiac diagnoses. A right-sided aortic arch was also commonly present in these children. Facial features varied and small ears were the most noteworthy anthropometric feature. A right-sided aortic arch with or without a congenital cardiac lesion, a long, narrow mid-face and small ears should alert the physician to the possibility of a microdeletion on the long arm of chromosome 22.

## Summary

Microdeletion of chromosome 22 at the q11 locus is the most common contiguous gene-deletion syndrome known to man and may be second only to Down syndrome in order of frequency. Prevalence estimates vary from one in 2 000 to one in 7 000, but it is generally accepted to have a prevalence of at least one in 4 000.^1^-^5^ It is clear therefore, that this disorder poses a significant health concern and clinicians should take note of this condition. The deleted region is identical in almost 90% of cases and consists of three million base pairs of DNA, containing a total of 32 genes.^4^

Nomenclature of this disorder has been confusing, most likely due to the enormous variability in phenotypic expression. It has been described using various names (e.g. DiGeorge sequence, Shprintzen syndrome). However, largely due to the dedication and work of Dr Robert Shprintzen, it is now generally known as the velocardiofacial syndrome (VCFS). To date, more than 180 phenotypic features have been described, although the major features of the syndrome are essentially abnormal facial characteristics as well as cardiac and palatal abnormalities.^4^,^6^-^8^

**Fig. 1.**
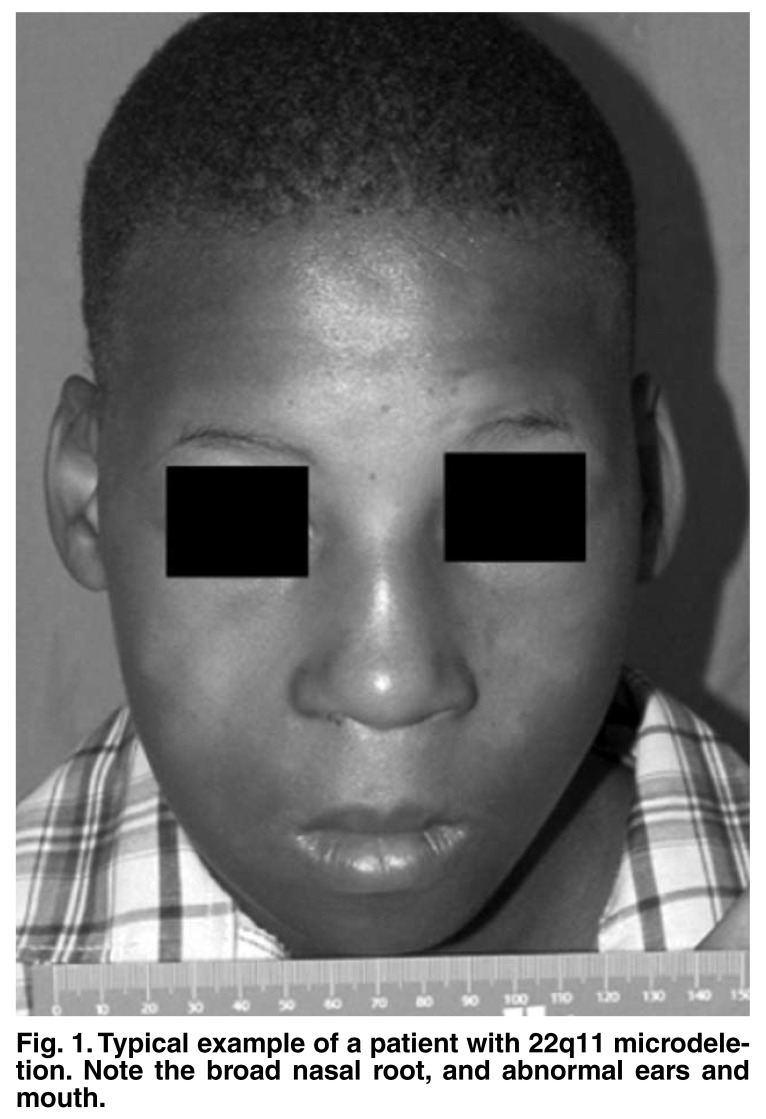
Typical example of a patient with 22q11 microdeletion. Note the broad nasal root, and abnormal ears and mouth.

Detectable cardiac abnormalities may be found in up to 75% of patients,^9^ and are largely responsible for the morbidity and mortality related to the syndrome.^10^ Facial dysmorphology varies significantly, with ‘classical’ features consisting of a prominent nasal root, abnormal ears and eyes, and a small mouth (Fig. 1). No feature is absolutely characteristic and this may lead to underrecognition of this condition. Furthermore, study populations predominantly consist of oriental children and those of European ancestry. In one study,^9^ a paucity of the features was recognised in African-American children, but they represented only 11% of the patients studied.

Little has been published regarding VCFS in South African children, especially among the indigenous population. The aim of this study was to determine the cardiac abnormalities as well as selected facial anthropometric measurements in affected children from the Free State and Northern Cape.

## Methods

The study was a prospective, descriptive investigation of children presenting at the Cardiology Unit of the Universitas Academic Hospital complex. All patients with one or more facial features suggestive of 22q11 microdeletion were included in this study. A positive fluorescence *in situ* hybridisation (FISH) analysis was required as proof of a microdeletion. The Vysis® LSI TUPLE 1 probe set containing the LSI TUPLE1 probe for chromosomal regions TUPLE1, D22S55, D22S609 and D22S942 with a LSI ARSA control probe was used (supplied by The Scientific Group, Johannesburg, SA).

Patient evaluation included a clinical examination as well as a routine complete paediatric echocardiogram. Echocardiography was performed using a Philips 5500 apparatus and appropriate transducers, using standard views. A cardiologist reviewed all the echocardiograms. Follow-up data were obtained from clinical records.

**Fig. 2.**
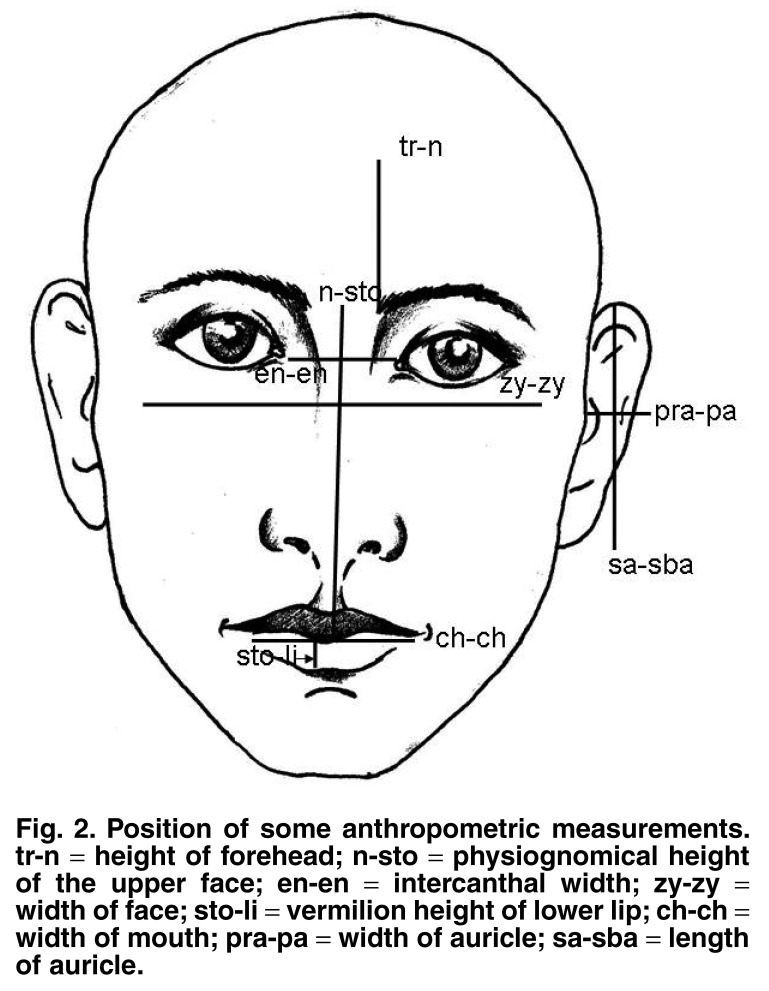
Position of some anthropometric measurements. tr-n = height of forehead; n-sto = physiognomical height of the upper face; en-en = intercanthal width; zy-zy = width of face; sto-li = vermilion height of lower lip; ch-ch = width of mouth; pra-pa = width of auricle; sa-sba = length of auricle.

Seventeen pre-selected standard craniofacial anthropometric measurements were performed using digital sliding callipers where possible. This leg of the study was started three years after the commencement of the initial trial. All measurements were performed as described by Farkas.^11^ One of the authors took all the measurements. For a more detailed description of the anthropometric measurements, see Fig. 2.

Both the initial and subsequent protocols were approved by the Ethics Committee of the Faculty of Health Sciences, University of the Free State (ETOVS 118/99). Written informed consent was obtained from the parent or legal guardian of the patient and verbal consent from the children as far as possible.

## Statistical analysis

Data were captured using Microsoft Excel spreadsheets and statistical analyses were performed by the Department of Biostatistics, University of the Free State, as well as with a commercially available software package, GraphPad Prism version 5.00 (GraphPad software, San Diego, California, USA). A *p*-value less than 0.05 was considered statistically significant, while 95% confidence intervals (CI) were used where clinically indicated. *Z*-values were obtained using a standard formula to compare anthropometric measurements with reference to a standard set of normal values.^11^

## Results

A total of 334 FISH analyses were done over an eight-year period (1999–2007), resulting in 40 patients being identified with the microdeletion. The median age at diagnosis (positive FISH probe) was 3.6 years with a range of 0.04 years (two weeks to 16.2 years). Twenty-one (52.5%) of the patients were male. The group consisted of 23 African and 10 Caucasian children, and seven children of mixed ethnic origin.

The primary echocardiographic findings are shown in Table 1. Tetralogy-type lesions were the most common (43%), followed by truncus arteriosus (20%). The miscellaneous group consisted of patent ductus arteriosus (*n* = 2), atrial septal defect (*n* = 2), double-outlet right ventricle (*n* = 1), and mitral valve atresia with hypoplastic left ventricle (*n* = 1). Right-sided aortic arches were present in 17 children (43%), either as an isolated finding or in combination with other congenital cardiac defects. Surgery was performed in 28 patients. Full repair was possible in 16 children and palliative procedures were carried out in 12. Low serum calcium was observed in six patients peri-operatively. Four patients died, one due to natural causes and three post-operatively. Death occurred three months to three years after surgery.

**Table 1:** Echocardiographic Findings

*Cardiac finding*	*Number of patients (n = 40)*	*%*
Tetralogy of Fallot	10	25.0
PA-VSD	7	17.5
Truncus arteriosus	8	20.0
VSD	4	10.0
Isolated right-sided aortic arch	2	5.0
Normal	3	7.5
Miscellaneous*	6	15.0

PA-VSD: pulmonary atresia with ventricular septal defect; VSD: ventricular septal defect. *See text for details.

Cleft palate was present in 32% (*n* = 14) of the patients. Skeletal abnormalities were present in two patients, with fusion of the radius and ulna in one patient and spina bifida in the other. One patient had psychiatric abnormalities as well as isolated upper motor neuron VII palsy. All children of school-going age in our study needed either remedial classes or special schooling. One patient was diagnosed with a T-cell deficiency and another with hypoparathyroidism.

Anthropometric measurements were obtained in 24 (60%) patients. Both height and weight for age were impaired compared to normal children, as reflected by median *z*-scores of –1.8 (–4 to 0), 95% CI: –1.3 to –2.2 for height, and –2 (–4.0 to 0.7), 95% CI: –1.4 to –2.6 for weight, respectively. Growth was analysed retrospectively using old records and height for age was compared after the longest periods of follow-up (median 12.7 years, range 3.9–19.7 years). The height for age increased from a median *z*-score of –3.4 (range –1.7 to –4.5) to –2.3 (range –2.1 to –3.2), with *p* = 0.08 and 95% CI: –1.7 to 0.2.

**Fig. 3.**
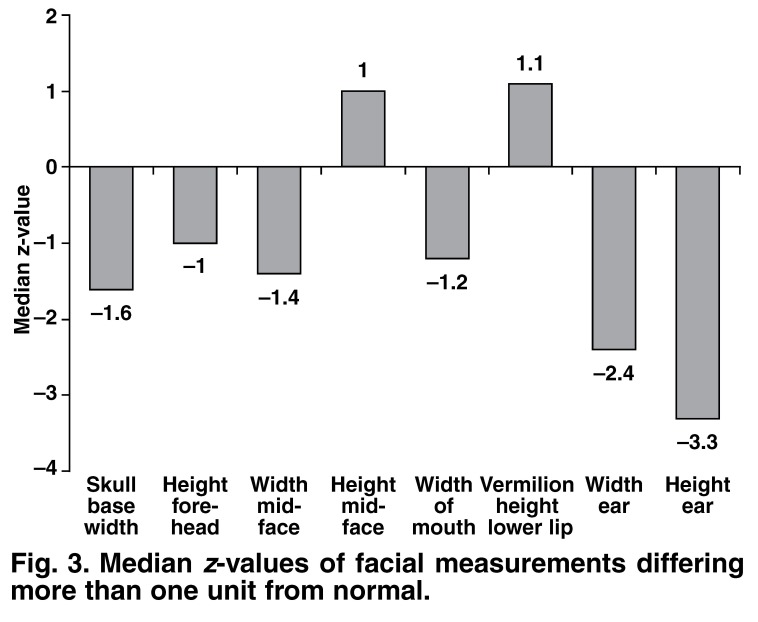
Median z-values of facial measurements differing more than one unit from normal.

Results for individual facial measurements are shown in Table 2, while Fig. 3 shows the median z-values of facial measurements differing from normal by a value of more than one. The median z-values for skull-base width, physiognomic height of upper face and width of mouth tended to be smaller than normal, while the vermilion height of the lower lip was somewhat higher than average. Both the height and width of the ears were notably smaller than normal, with median z-scores of –3.3 and –2.4, respectively (Table 1). A right-sided aortic arch was present in 52% (10/19) of the children with a z-value of less than –2 for ear height (sensitivity 0.2; specificity 0.5).

**Table 2:** Anthropometric Measurements Of The Face

	*z-value*			
*Description*	*Median*	*25th %*	*75th %*	*95% CI*
Skull-base width (t-t)	–1.6	–2.9	–0.5	(–2.5; –1.1)
Height of forehead (tr-n)	–1.0	–1.7	0.4	(–1.3; –0.2)
Height of mid-face (n-sto)	1.0	–0.5	3.3	(0.0; 2.5)
Width of face (zy-zy)	–1.4	–2.2	0.1	(–2.1; –0.5)
Width of mandible (go-go)	0.8	–0.6	1.9	(–2.3; 1.3)
Intercanthal width (en-en)	–0.5	–1.7	1.0	(–1.2; 0.6)
Bi-occular width (ex-ex)	–0.2	–0.9	1.9	(–2.3; 1.3)
Width of nose (al-al)	0.7	–1.2	2.0	(–0.4; 1.3)
Height of nose (n-sn)	1.0	0.0	2.6	(0.5; 2.4)
Nasal tip protrusion (sn-prn)	–0.7	–2.3	0.6	(–2.1; 0.4)
Width of columella (sn’-sn’)	0.5	–1.0	2.1	(–0.2; 1.9)
Length of ala (ac-prn)	–0.8	–2.5	0.8	(–1.9; 1.0)
Width of mouth (ch-ch)	–1.2	–2.3	–0.3	(–1.8; –0.3)
Vermilion height of lower lip (sto-li)	1.1	–0.7	2.2	(0.1; 1.6)
Width of auricle (pra-pa)*	–2.4	–3.4	–1.4	(–3.3; –1.7)
Height of ear (sa-sba)*	–3.3	–4.8	–2.6	(–4.5; –2.9)

*The width and height of ears reflects the average of both ears.

## Discussion

To the authors’ best knowledge, this is the first published report on cardiac and facial features of the 22q11 microdeletion in South African children. Our results show that microdeletions of the long arm of chromosome 22 occur in children from the Free State and Northern Cape provinces. Cyanotic heart lesions were common, especially conotruncal defects. Right-sided aortic arches were frequently found either in isolation or associated with underlying cardiac lesions. Anthropometric abnormalities, especially a long, narrow mid-facial area and small ears were the most marked facial anomalies.

Cardiac abnormalities were present in 92% of our patients, but the fact that most of these patients presented at a cardiology unit should be taken into account. Cardiac abnormalities are common and occur in up to 75% of children with VCFS.^9^ Tetralogy of Fallot, pulmonary atresia with ventricular septal defect (VSD) (also referred to as extreme tetralogy) and truncus arteriosus were present in 63% of our patients. This compares favourably with international studies where tetralogy, with or without pulmonary atresia,^12^–^17^ were also the most common cardiac lesions. These are all cyanotic cardiac conditions and are often complex lesions requiring early and multiple surgical interventions. This is highlighted by the fact that 75% (28/37) of our patients required surgery, of which 42% (12/28) were palliative surgical procedures.

Three patients died post-operatively and another one died totally unrelated to surgery. This suggests that mortality in these patients does not differ markedly from non-syndromic children with similar congenital cardiac lesions. In our opinion, the presence of VCFS should therefore not delay or deter cardiac surgery. Acceptable surgical outcomes are supported by other studies, but the complexity of the underlying lesion and associated anomalies should be taken into account.^18^-^21^

A common cardiac finding in our patient group was a right-sided aortic arch, which was present in almost half of the children, compared to the 0.1% in a normal patient population.^22^,^23^ This is of clinical importance and therefore one should consider the presence of microdeletions of chromosome 22q11 in patients with right-sided aortic arches. It is also noteworthy that six patients presented post-operatively with low serum calcium values. Hypocalcaemia has been described in 17 to 50% of VCFS cases.^5^,^10^,^24^ Cardiothoracic surgeons and intensivists taking care of these patients should be aware of this potential problem, since it may lead to seizures.

The age of our patients at diagnosis was fairly advanced, essentially due to the fact that we tested previously-seen as well as new patients at the clinic only once the laboratory introduced testing with the FISH probe. As the investigators became familiar with the facial features, the diagnosis was made at a much earlier age, even before two weeks of age.

Short stature was common in our patients, but it is an interesting finding that these children had a mild, although not statistically significant, improvement in growth as they got older. This finding lends further evidence to other studies, which showed that features differ in older patients^25^,^26^ and that the microdeletion 22q11 phenotype may evolve with time. Studies with objective anthropometric measurements of facial characteristics in children with VCFS are rare.^26^

Our results mostly showed minor abnormal facial measurements. Small, narrow ears were the most notable finding. However, this is also found in other syndromes, for example Down syndrome. A narrow face and a longish mid-face were the other more prominent abnormal measurements compared to normal reference values. These findings are in agreement with literature reports and confirm that facial abnormalities are varying and inconsistent features in children with VCFS.^26^–^28^ Alternatively, these findings could support the results of McDonald-McGinn^9^ that facial features in African children are less pronounced, since they made up 70% (17/24) of the group in which we performed facial measurements.

Overall, the results of this study are in agreement with international data. It is clear, therefore, that South African physicians should take note of this condition. Apart from the fact that cardiac defects are common and an important cause of morbidity and mortality, the associated problems should be recognised and managed. Developmental delay, failure to thrive and feeding difficulties are common during infancy.^27^,^28^ Forty-five per cent of children with the microdeletion have conductive hearing loss or hypernasal speech (75%) and these are some of the most distressing aspects for parents.^27^,^29^,^30^

Furthermore, behavioural problems such as attention deficit disorder, autistic spectrum disorder, bipolar disorder and schizophrenia have been reported in 10 to 30% of teenagers and adults with VCSF.^31^,^32^ Learning difficulties were universal in the children of school-going age in this study and early diagnosis and intervention can reduce the impact. This syndrome is therefore important to recognise since these children need appropriate referral and specialised, multidisciplinary care for their multiple medical, learning and social problems.

## Limitations of the study

The patients were a selected group due to the fact that the study was confined to children presenting at a cardiology unit and it is therefore not a reflection of the prevalence of this syndrome. The study was also limited to the more commonly abnormal measurable facial features and did not attempt to describe all the facial dysmorphic features of the 22q11 microdeletion syndrome. There are no normal values for the facial measurements of South African children and the mean values used to determine the z-scores are for American children. It is clear that further studies are indicated to determine the prevalence and long-term outcome of VCFS in South African children.

## Conclusions

Microdeletions of chromosome 22q11 are present in children from the Free State and Northern Cape. Conotruncal cyanotic heart lesions, especially tetralogy with or without pulmonary atresia and truncus arteriosus were the most frequent congenital cardiac diagnoses. A right-sided aortic arch was commonly present in these children. Facial features varied and small ears were the most notable anthropometric feature. A right-sided aortic arch with or without a congenital cardiac lesion, long, narrow mid-face and small ears should alert the physician to the possibility of a microdeletion on the long arm of chromosome 22 and prompt FISH probe testing.
